# Modified minimum principal stress estimation formula based on Hoek–Brown criterion and equivalent Mohr–Coulomb strength parameters

**DOI:** 10.1038/s41598-023-33053-x

**Published:** 2023-04-19

**Authors:** Yanhui Song, Man Feng, Peng Chen

**Affiliations:** 1 School of Geology Engineering and Geometrics, Chang’an University, Xi’an, 710054 China; 2 JiNan Design Institute of China Railway Engineering Design and Consulting Co. LTD, Jinan, 250022 Shandong China

**Keywords:** Natural hazards, Civil engineering

## Abstract

The most critical parameter for determining equivalent values for the Mohr–Coulomb friction angle and cohesion from the nonlinear Hoek–Brown criterion is the upper limit of confining stress. For rock slopes, this value is the maximum value of the minimum principal stress ($\sigma_{3,\max }^{\prime }$) on the potential failure surface. The existing problems in the existing research are analyzed and summarized. Using the finite element method (FEM), the location of potential failure surfaces for a wide range of slope geometries and rock mass properties are calculated using the strength reduction method, and a corresponding finite element elastic stress analysis was carried in order to determine $\sigma_{3,\max }^{\prime }$ of the failure surface. Through a systematic analysis of 425 different slopes, it is found that slope angle (*β*) and geological strength index (GSI) have the most significant influence on $\sigma_{3,\max }^{\prime }$ while the influence of intact rock strength and the material constant $m_{i}$ are relatively small. According to the variation of $\sigma_{3,\max }^{\prime }$ with different factors, two new formulas for estimating $\sigma_{3,\max }^{\prime }$ are proposed. Finally, the proposed two equations were applied to 31 real case studies to illustrate the applicability and validity.

## Introduction

At present, limit equilibrium method based on Mohr–Coulomb (MC) failure criterion is still the main method for slope stability analysis. However, some studies show that the nonlinear Hoek–Brown (HB) failure criterion more correctly represents rock failure for almost all rock types^1–5^. Multiple methods for evaluating the equivalent MC friction angle and cohesion have been proposed^5–15^. Iamael and Konietxky^16^ modified the HB criterion to consider the anisotropy of rock by applying an explicit function of the rock parameter *m*_*i*_ with orientation *β*.

The Hoek–Brown criterion was firstly proposed for intact rock by Hoek and Brown in 1980^17^, and the latest version for rock mass is as follows^5^:

1 $$\sigma_{1} = \sigma_{3} + \sigma_{ci} \left( {m_{b} \frac{{\sigma_{3} }}{{\sigma_{ci} }} + s} \right)^{a}$$

where $\sigma_{1}$ and $\sigma_{3}$ are the major and minor principal stresses, $\sigma_{ci}$ is the unconfined compressive strength*,* and *m*_*b*_, *s*, and *a* are rock mass material constants given by Eqs. (2), (3), and (4), respectively.

2 $$m_{b} = m_{i} \exp \left( {\frac{GSI - 100}{{28 - 14D}}} \right)$$

3 $$s = \exp \left( {\frac{GSI - 100}{{9 - 3D}}} \right)$$

4 $$a = \frac{1}{2} + \frac{1}{6}\left( {e^{{ - \frac{GSI}{{15}}}} - e^{{ - \frac{20}{3}}} } \right)$$

where $m_{i}$ is a material constant for intact rock, GSI is the geological strength index which depends on rock mass characterization and commonly varies from 0 to 100; D is a factor which depends upon the degree of disturbance due to blast damage and stress relaxation and varies from 0 to 1.

The GSI classification system is based upon the assumption that the rock mass contains sufficient number of ‘randomly’ oriented discontinuities such that it behaves as an isotropic mass. Therefore, the control failure of a single discontinuous structure is beyond its range, which will lead to highly anisotropic mechanical behavior.

In line with the above discussion, it is important to realise the research in this paper will be subject to the same limitations that underpin the Hoek–Brown yield criterion itself.

Hoek^9^ proposed a method to calculate the equivalent Mohr–Coulomb parameters based on instantaneous rock mass properties for: (1) a specified effective normal stress, (2) a specified minor principal effective stress, and (3) a condition in which the rock mass uniaxial compressive strength is the same for both the Hoek–Brown and Mohr–Coulomb criteria. In 1997, Hoek and Brown^8^ revised the method of calculating the equivalent Mohr–Coulomb parameters according to the generalized Hoek–Brown criterion. It is recommended that the maximum value of the minimum effective principal stress generally be 0.25$\sigma_{ci}$, and the estimated *c* value using this method be decreased by 25% to avoid overestimating the rock mass strength. Also, for rock slopes, the effective normal stress on the potential failure surface of the slope may be small, so the maximum value of the minimum effective principal stress 0.25$\sigma_{ci}$ should be applied cautiously, otherwise the rock mass shear strength mass may be overestimated. For rock slopes, a minor principal stress range of 0 < *σ*_3_ < *σ*_*v*_ can be used, where *σ*_*v*_ = depth × unit weight of the rock mass^18^. In this case, depth is defined as the average depth of a failure surface in which a circular type can be assumed.

The equations for determining the equivalent cohesion and friction angle proposed by Hoek et al. in 2002 and 2018 are^5,19^:

5 $$c^{\prime } = \frac{{\sigma_{ci} \left[ {\left( {1 + 2a} \right)s + \left( {1 - a} \right)m_{b} \sigma_{3n}^{\prime } } \right]\left( {s + m_{b} \sigma_{3n}^{\prime } } \right)^{a - 1} }}{{\left( {1 + a} \right)\left( {2 + a} \right)\sqrt {1 + \left( {6am_{b} \left( {s + m_{b} \sigma_{3n}^{\prime } } \right)^{a - 1} } \right)/\left( {1 + a} \right)\left( {2 + a} \right)} }}$$

6 $$\phi \prime = \sin^{ - 1} \left[ {\frac{{6am_{b} \left( {s + m_{b} \sigma_{3n}^{\prime } } \right)^{a - 1} }}{{2\left( {1 + a} \right)\left( {2 + a} \right) + 6am_{b} \left( {s + m_{b} \sigma_{3n}^{\prime } } \right)^{a - 1} }}} \right]$$

with

7 $$\sigma_{3n}^{\prime } = \frac{{\sigma_{3\max }^{\prime } }}{{\sigma_{ci} }}$$

8 $$\frac{{\sigma_{3\max }^{\prime } }}{{\sigma_{cm} }} = 0.72\left( {\frac{{\sigma_{cm} }}{\gamma H}} \right)^{ - 0.91}$$

where γ is the rock mass unit weight, H is the slope height, and $\sigma_{cm}$ is the rock mass global strength, which is expressed as follows:

9 $$\sigma_{cm} = \sigma_{ci} \frac{{\left[ {m_{b} + 4s - a\left( {m_{b} - 8s} \right)} \right]\left( {\frac{{m_{b} }}{4} + s} \right)^{a - 1} }}{{\left( {1 + a} \right)\left( {2 + a} \right)}}$$

Li et al.^11^ found that, for steep slopes (i.e. greater than 45°), the safety factors calculated using the equivalent friction angle and cohesive strength obtained from Eqs. (5–9) are significantly higher due to the deviation of the estimated $\sigma_{3,\max }^{\prime }$; therefore, they suggested the following modified power functions to estimate $\sigma_{3,\max }^{\prime }$:

10 $$\frac{{\sigma_{3\max }^{\prime } }}{{\sigma_{cm} }} = 0.41\left( {\frac{{\sigma_{cm} }}{\gamma H}} \right)^{ - 1.23} \,{\text{for}}\,\beta \, < \,{45}^\circ$$

11 $$\frac{{\sigma_{3\max }^{\prime } }}{{\sigma_{cm} }} = 0.2\left( {\frac{{\sigma_{cm} }}{\gamma H}} \right)^{ - 1.07} \,{\text{for}}\,\beta \, \ge \,{45}^\circ$$

where *β*is the slope angle.

Renani and Martin^13^ also studied the estimation of $\sigma_{3,\max }^{\prime }$ through systematic slope stability analysis and found that using the $\sigma_{3,\max }^{\prime }$ calculated from Eq. (8) resulted in a 14% overestimation of the safety factor on average with higher discrepancies for steeper slopes, which more importantly led to drastic overestimation of the normalized failure area by an average of 79%. And what is more, they found that $\frac{{\sigma_{3,\max }^{\prime } }}{\gamma H}$ is almost independent of $\frac{{\sigma_{cm} }}{\gamma H}$ and is primarily controlled by the slope angle; therefore, Renani and Martin^13^ proposed the following equation to estimate $\sigma_{3,\max }^{\prime }$:

12 $$\frac{{\sigma_{3\max }^{\prime } }}{\gamma H} = \frac{0.175}{{\tan \left( \beta \right)}}$$

Equation (12) is obtained from an analysis of slopes with a range of parameters (Table 1).*β*, *m*_*i*_, GSI, and D almost cover the whole range of possible values (Table 1). Only $\frac{{\sigma_{ci} }}{\gamma H}$ covers a narrow range of possible values. For example, when slope height H = 100 m, and γ = 0.027 MN/m^3^, then $\sigma_{ci}$ = 0.27–27 MPa, this situation represents only a small portion of natural rock slopes, therefore when $\frac{{\sigma_{ci} }}{\gamma H} > 10$, the applicability of Eq. (12) needs to be verified.

**Table 1:** Range of slope parameters.

Parameter	*β*(°)	$\frac{{\sigma_{ci} }}{\gamma H}$	*m*_*i*_	GSI	D
Minimum	30	0.1	5	20	0
Maximum	75	10	30	80	1

The magnitude of the minimum principal stress on the potential failure surface of the rock slope is primarily related to its development location, which is not only related to slope angle but also to the intact rock strength and rock mass integrity. However, Eq. (8) does not consider the influence of slope angle and Eq. (12) does not take into account the effect of the intact rock strength and rock mass integrity. Although all factors are considered in Eqs. (10) and (11), the slope angle is divided into two cases: less than 45°and greater than or equal to 45°, which fails to consider the effect of a continuously changing slope angle on the minimum principal stress magnitude. In view of these problems that still exist in the current research, this contribution aims to propose a new estimation formula for $\sigma_{3,\max }^{\prime }$ on the potential failure surface of the slope by extending the range of $\frac{{\sigma_{ci} }}{\gamma H} > 10$ in Table 1.

## Methodology

In order to establish the estimation formula of $\sigma_{3,\max }^{\prime }$ on the slope potential failure surface, the finite element strength reduction method for generalized Hoek–Brown criterion was adopted to calculate the location of the potential failure surface for a wide range of slope geometries and rock mass properties. Then the corresponding finite element elastic stress analysis was carried out in order to determine the value of $\sigma_{3,\max }^{\prime }$ on the failure surface (based on the method proposed by Renani and Martin in^13^). The $\sigma_{3,\max }^{\prime }$ values on 425 potential slope failure surfaces are calculated and used in a statistical analysis to obtain new estimation formulas.

It is noteworthy to highlight that in reality, even for the rock mass exhibits isotropic characteristics, the presence of distinct structural planes and fault can lead to deviations in the sliding surface of local slopes. However, these deviations are generally considered to be within acceptable limits.

**Figure 1:**
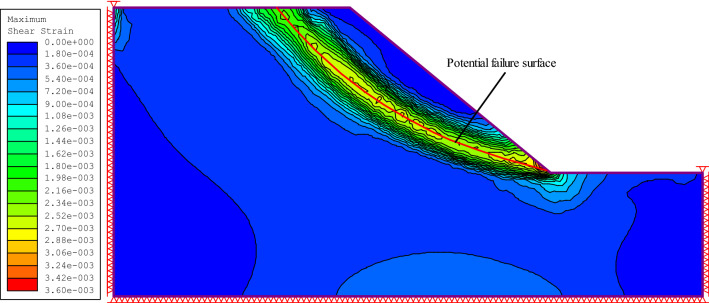
Potential failure surface calculated using the strength reduction method.

**Figure 2:**
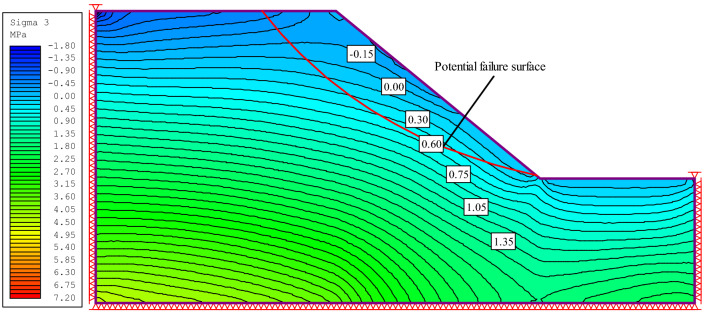
$\sigma_{3,\max }^{\prime }$ on the potential failure surface**.**

Table 2 shows the range of slope parameters used in this study. Finite element strength reduction analysis and elastic stress analysis was carried out using RS2 software. The gravitational stress field had a horizontal to vertical in situ stress ratio of unity, rock mass deformation modulus $E_{rm}$ was estimated using the Eq. (13)^20^, the rock mass residual index is the same as the peak index, and Poisson’s ratio was 0.28. Figure 1 shows the position and shape of the potential failure surface of the slope calculated using the finite element strength reduction method (corresponding to the maximum shear strain band), and Fig. 2 shows the minimum principal stress $\sigma_{3,\max }^{\prime }$ on the potential failure surface.

13 $$E_{rm} = 100000\left( {\frac{1 - D/2}{{1 + e^{{\left( {\left( {75 + 25D - GSI} \right)/11} \right)}} }}} \right)$$

**Table 2:** Range of slope parameters in this study.

Parameter	*β*(°)	$\frac{{\sigma_{ci} }}{\gamma H}$	*m*_*i*_	GSI	D
Minimum	30	10	5	20	0
Maximum	70	50	30	80

The disturbance factor D was not considered in this study because the disturbed zone of the slope caused by blasting excavation is primarily limited to the shallow part of the slope; hence, D should not be used for the entire slope^19^. Due to the difference in blasting methods, slope shapes, and rock mechanical properties, the range of slope disturbance zones varies significantly. At present, there is no method to estimate this range, which makes considering the effect of D difficult.

The disturbance factor D is usually considered to take into account of the effects of reduction of GSI caused by construction disturbance. When the slope is analyzed for various GSI values, the individual effect of D can be safely ignored.

## Impact analysis of various factors

**Figure 3:**
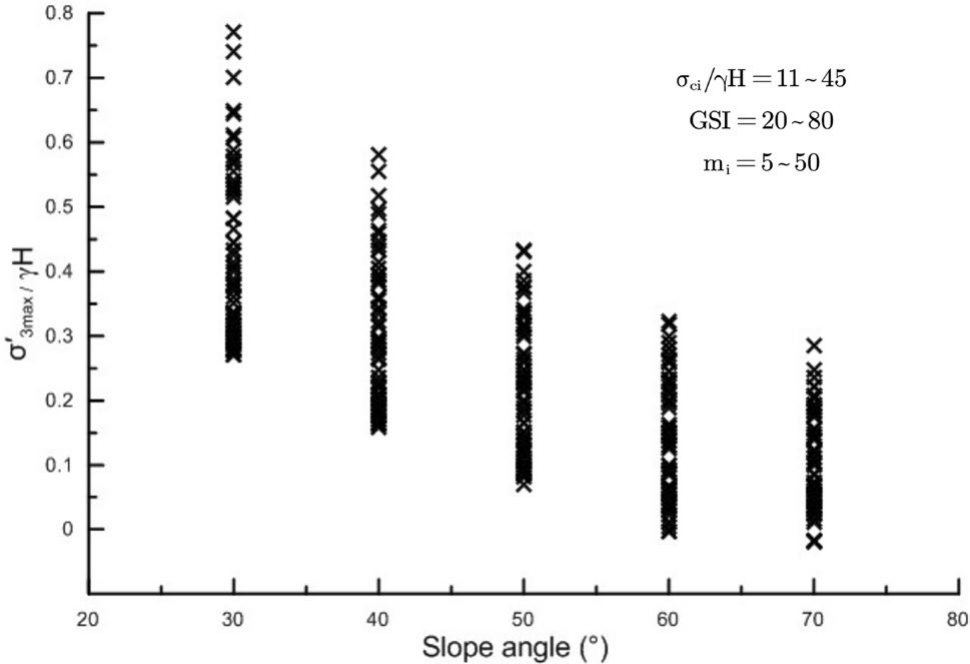
Change in $\frac{{\sigma_{3,\max }^{\prime } }}{\gamma H}$ for various slope angles.

As mentioned earlier, Eq. (8) does not consider the influence of slope angle, while Eq. (12) only considers the influence of slope angle without considering the intact rock strength and rock mass integrity. This study shows that for slopes with varying rock mass properties, there will be a large difference range of $\sigma_{3,\max }^{\prime }$ when the range of $\frac{{\sigma_{ci} }}{\gamma H}$ is between 10 and 50, even for the same slope angles. When the slope angle is 30°, the difference in $\frac{{\sigma_{3,\max }^{\prime } }}{\gamma H}$ can reach 0.5 (Fig. 3), indicating that in addition to the slope angle, the rock mass properties have a non-negligible impact on $\sigma_{3,\max }^{\prime }$.

### $\sigma_{ci}$to γH ratio

**Figure 4:**
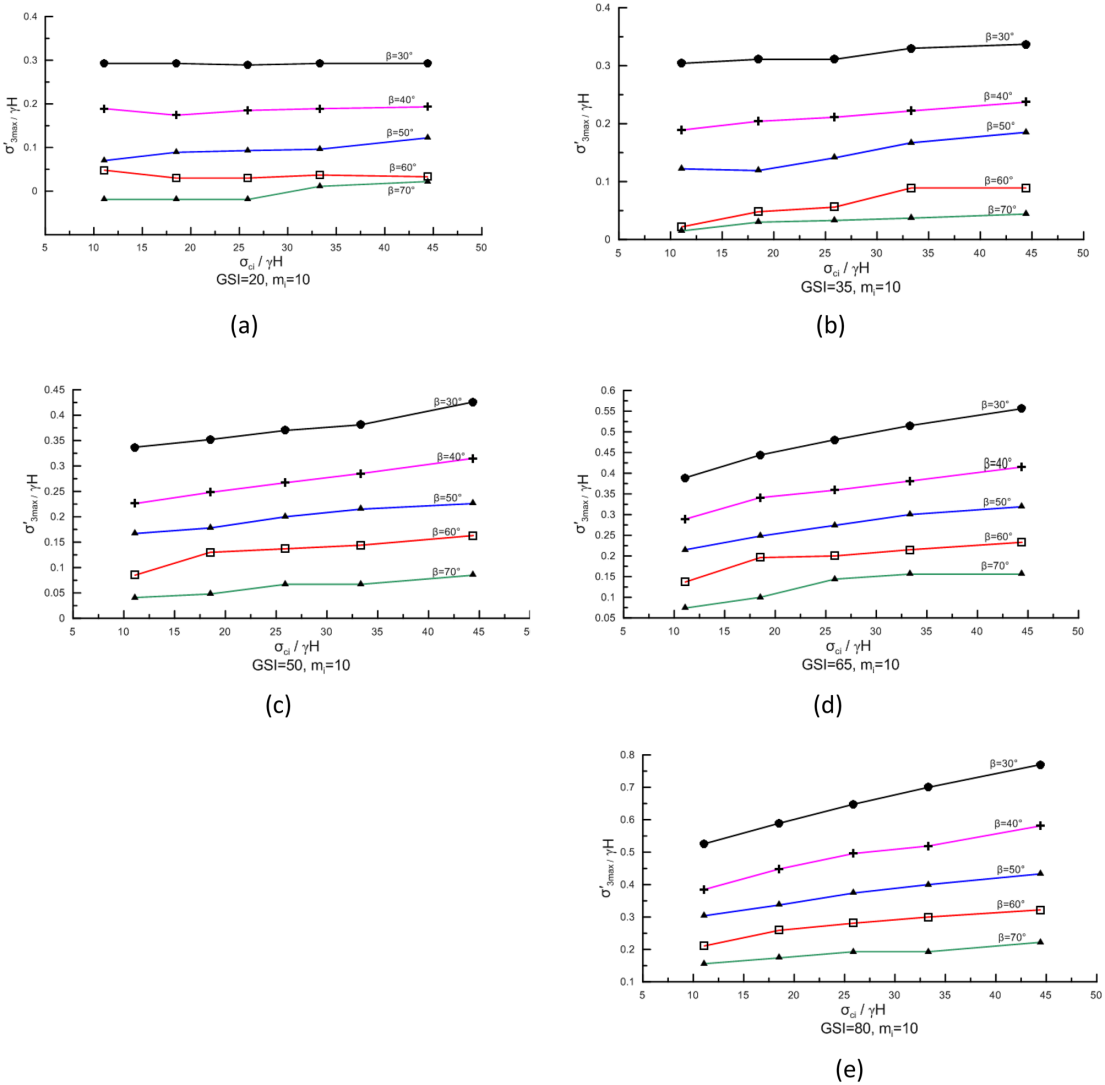
Correlation between $\frac{{\sigma_{3,\max }^{\prime } }}{\gamma H}$ and $\frac{{\sigma_{{{\text{ci}}}} }}{\gamma H}$.

Homogeneous rock slope failure is related to the strength of the intact rock that composes the slope. Under the same conditions, the greater the uniaxial compressive strength of the rock block, the higher the slope stability and the deeper the potential failure surface. In this study, $\frac{{\sigma_{ci} }}{\gamma H}$ is used to characterize the effect of rock block strength on $\sigma_{3,\max }^{\prime }$ of the potential failure surface. Figure 4a–e show the correlation between $\frac{{\sigma_{3,\max }^{\prime } }}{\gamma H}$ and $\frac{{\sigma_{ci} }}{\gamma H}$ when GSI = 20, 35, 50, 65, and 80, respectively.

When GSI is small (i.e., GSI < 50), no matter the slope angle, $\frac{{\sigma_{ci} }}{\gamma H}$ has little effect on $\frac{{\sigma_{3,\max }^{\prime } }}{\gamma H}$; when GSI is large (i.e., GSI ≥ 50), the magnitude of $\frac{{\sigma_{3,\max }^{\prime } }}{\gamma H}$ increases slowly with increasing $\frac{{\sigma_{ci} }}{\gamma H}$, and the smaller the slope angle, the greater the increase (Fig. 4).

### Geological Strength Index (GSI)

**Figure 5:**
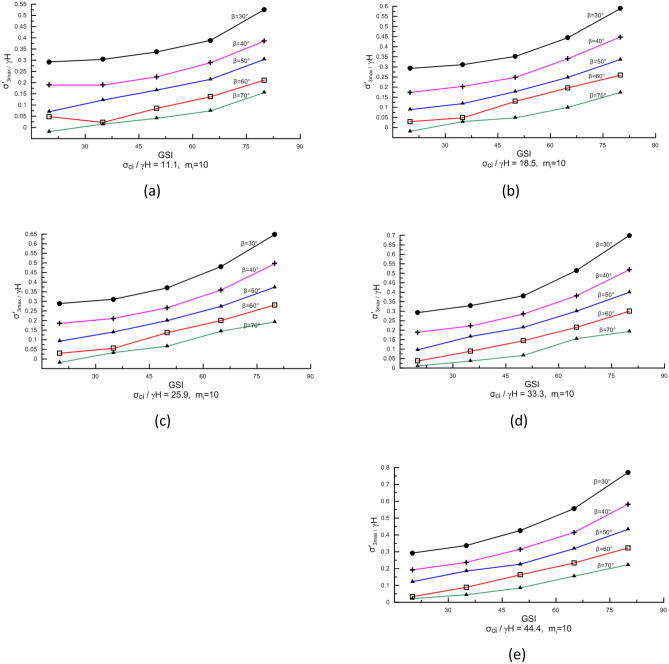
Correlation between $\frac{{\sigma_{3,\max }^{\prime } }}{\gamma H}$ and GSI.

The Geological Strength Index (GSI) reflects rock mass integrity and is the most important factor affecting slope stability and the location of the potential failure surface. GSI has a significant impact on the magnitude of $\sigma_{3,\max }^{\prime }$ on the potential failure surface. Figure 5a–e show the correlation between $\frac{{\sigma_{3,\max }^{\prime } }}{\gamma H}$ and GSI when $\frac{{\sigma_{ci} }}{\gamma H}$ ranges from 11 to 45.

$\frac{{\sigma_{3,\max }^{\prime } }}{\gamma H}$ values increase exponentially with increasing GSI value at different slope angles, and the growth curves at different slope angles are basically parallel, indicating that the change in $\frac{{\sigma_{3,\max }^{\prime } }}{\gamma H}$ with GSI is not affected by the size of the slope angle (Fig. 5). Similarly, the change in $\frac{{\sigma_{3,\max }^{\prime } }}{\gamma H}$ with GSI is also not affected by $\frac{{\sigma_{ci} }}{\gamma H}$ (Fig. 5a–e).

### Material constant ***m***_***i***_

$m_{i}$ is a material constant for the intact rock which depends upon the mineralogy, composition, and grain size of the intact rock^21^, which is obtained from laboratory. Hoek and Brown^19^ proposed an approximate relationship between the compressive to tensile strength ratio,$\frac{{\sigma_{ci} }}{{\left| {\sigma_{t} } \right|}}$, and the Hoek–Brown parameter *m*_*i*_:

14 $$\frac{{\sigma_{ci} }}{{\left| {\sigma_{t} } \right|}} = 0.81m_{i} + 7$$

where $\left| {\sigma_{t} } \right|$ is the absolute value of the uniaxial tensile strength.

**Figure 6:**
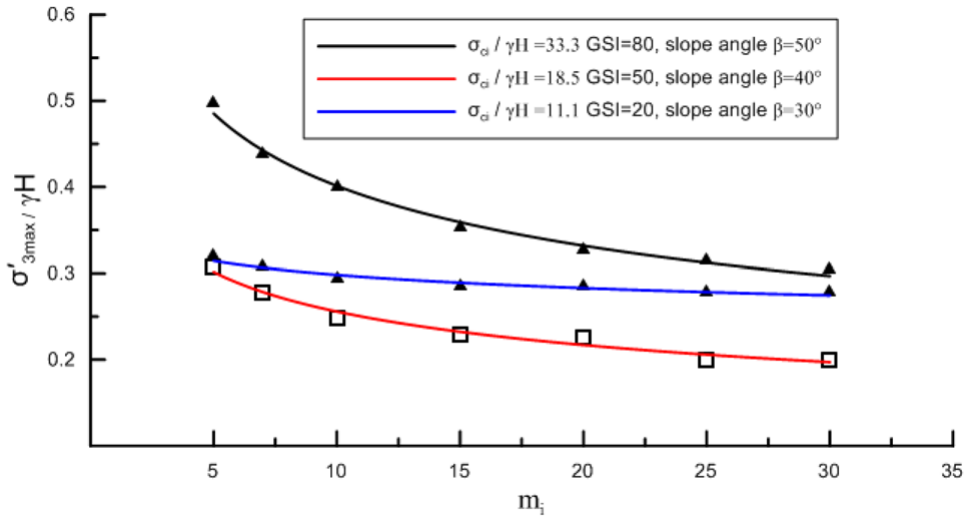
Effect of *m*_*i*_ on $\frac{{\sigma_{3,\max }^{\prime } }}{\gamma H}$.

In order to examine the influence of $m_{i}$ on $\frac{{\sigma_{3,\max }^{\prime } }}{\gamma H}$ on the potential failure surface, the variation law of $\frac{{\sigma_{3,\max }^{\prime } }}{\gamma H}$ with $m_{i}$ for varying slope angles and rock mass parameters is calculated and analyzed. Results show that, $\frac{{\sigma_{3,\max }^{\prime } }}{\gamma H}$ always decreases as a power function with increasing *m*_*i*_, and the decrease range is commonly small. For example, when $m_{i}$ varies from 5 to 30, the difference in $\frac{{\sigma_{3,\max }^{\prime } }}{\gamma H}$ is within 0.2, indicating that $m_{i}$ has no significant effect on $\frac{{\sigma_{3,\max }^{\prime } }}{\gamma H}$. Especially when *m*_*i*_ > 10, its effect on $\frac{{\sigma_{3,\max }^{\prime } }}{\gamma H}$ is very small. Figure 6 shows the variation of $\frac{{\sigma_{3,\max }^{\prime } }}{\gamma H}$ on potential failure surface with $m_{i}$ under three different conditions.

### Slope angle

**Figure 7:**
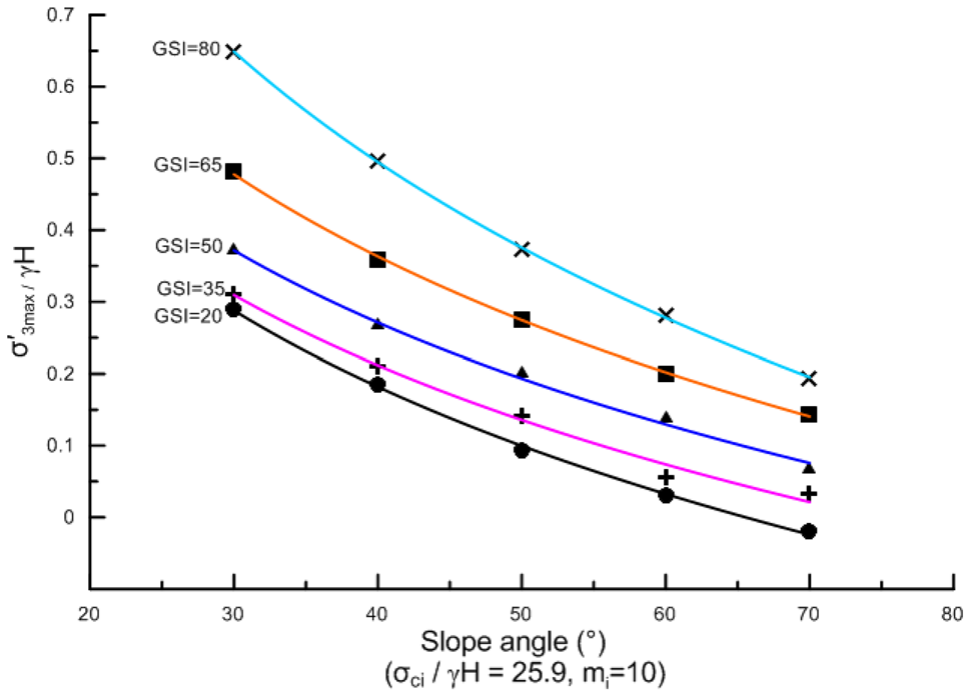
Variation of $\frac{{\sigma_{3,\max }^{\prime } }}{\gamma H}$ with slope angle.

Slope angle not only affects the stress distribution in the rock mass but also affects the location of the potential failure surface, indicating that the effect of slope angle on $\sigma_{3,\max }^{\prime }$ is significant. Numerous calculations show that when other conditions are the same, $\sigma_{3,\max }^{\prime }$ decreases exponentially with increasing slope angle. Figure 7 shows the variation of $\frac{{\sigma_{3,\max }^{\prime } }}{\gamma H}$ with slope angle when $\frac{{\sigma_{ci} }}{\gamma H}$ = 25.9, $m_{i}$ = 10, and GSI is 20, 35, 50, 65, and 80, respectively. In other cases, the variation of $\frac{{\sigma_{3,\max }^{\prime } }}{\gamma H}$ with slope angle follows the same law.

## New equation for estimating appropriate range of confining stress

In "Impact analysis of various factors", the influence of various factors on $\sigma_{3,\max }^{\prime }$ of the potential failure surface is analyzed. Results show that slope angle and GSI have the most significant influence on $\sigma_{3,\max }^{\prime }$, followed by the intact rock strength, while the $m_{i}$ has the least effect. According to the variation pattern of $\sigma_{3,\max }^{\prime }$ with each factor, the following function is used as a fitting function:

15 $$\frac{{\sigma_{3\max }^{\prime } }}{\gamma H} = b\frac{{\left( {\frac{{\sigma_{ci} }}{\gamma H}} \right)^{c} e^{\frac{GSI}{k}} }}{{m_{i}^{d} \left( {\tan \beta } \right)^{f} }} + t$$

where b, c, k, d, f, and t are constants.

Based on the above assumptions, an analysis was carried out on the calculation results of 425 slopes with a wide range of slope geometries and rock mass properties. Equation (15) was fit to these data, and the following best-fit equation was derived:

16 $$\frac{{\sigma_{3\max }^{\prime } }}{\gamma H} = \frac{{0.173\left( {\frac{{\sigma_{ci} }}{\gamma H}} \right)^{0.165} e^{{\frac{GSI}{{75.2}}}} }}{{m_{i}^{0.219} \left( {\tan \beta } \right)^{0.631} }} - 0.1\,{\text{R}}^{{2}} \, = \,0.{953}$$

**Figure 8:**
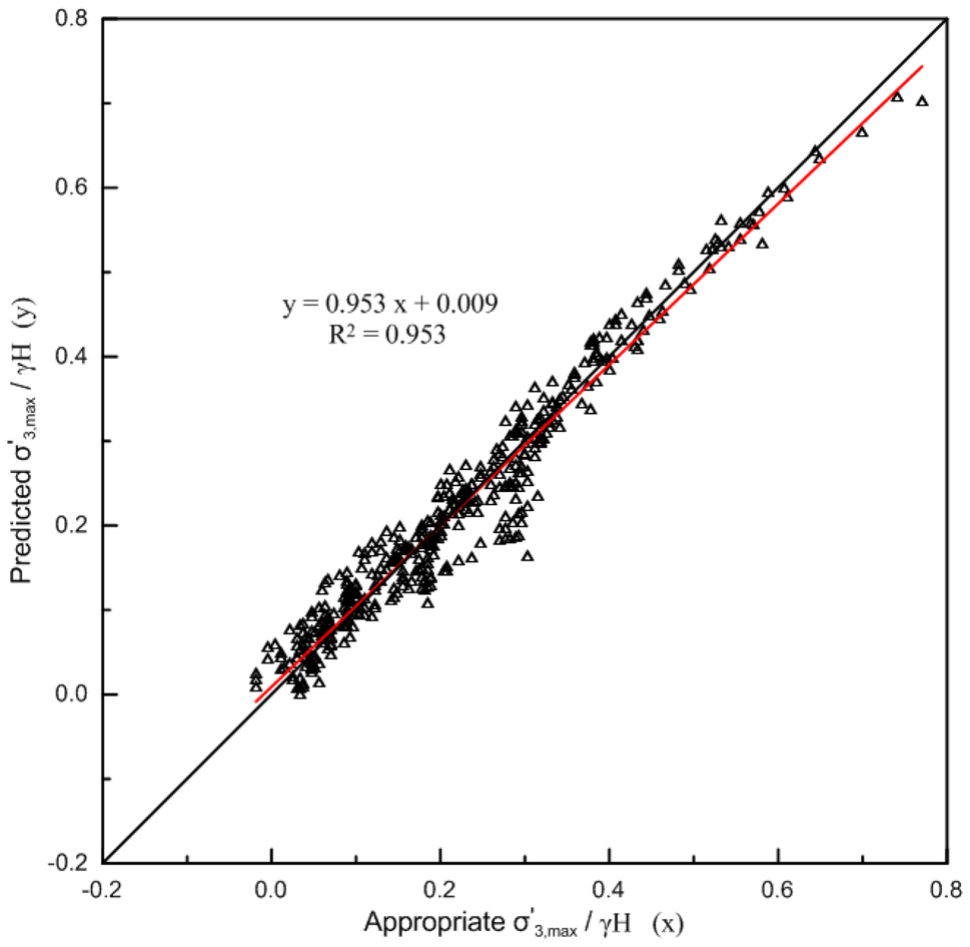
Comparison between appropriate $\frac{{\sigma_{3,\max }^{\prime } }}{\gamma H}$ values and predicted values from Eq. (16).

The $\frac{{\sigma_{3,\max }^{\prime } }}{\gamma H}$ values predicted from Eq. (16) are plotted against appropriate values (finite element method results) in Fig. 8. The correlation between the predicted and appropriate $\frac{{\sigma_{3,\max }^{\prime } }}{\gamma H}$ values is reasonably close to the ideal 1:1 relationship of a perfect fit.

Although the fitting degree of Eq. (16) is sufficiently accurate, its expression is cumbersome. In order to simplify Eq. (16), $\frac{{\sigma_{ci} }}{\gamma H}$ and $m_{i}$ are removed from Eq. (16) considering their minor influence on $\sigma_{3,\max }^{\prime }$, and a value of f = 1 is adopted. On this basis, the same analysis was completed, and a simplified estimation formula for $\sigma_{3,\max }^{\prime }$ was obtained:

17 $$\frac{{\sigma_{3\max }^{\prime } }}{\gamma H} = \frac{{0.1e^{0.017GSI} }}{\tan \beta }\,{\text{R}}^{{2}} \, = \,0.{89}$$

**Figure 9:**
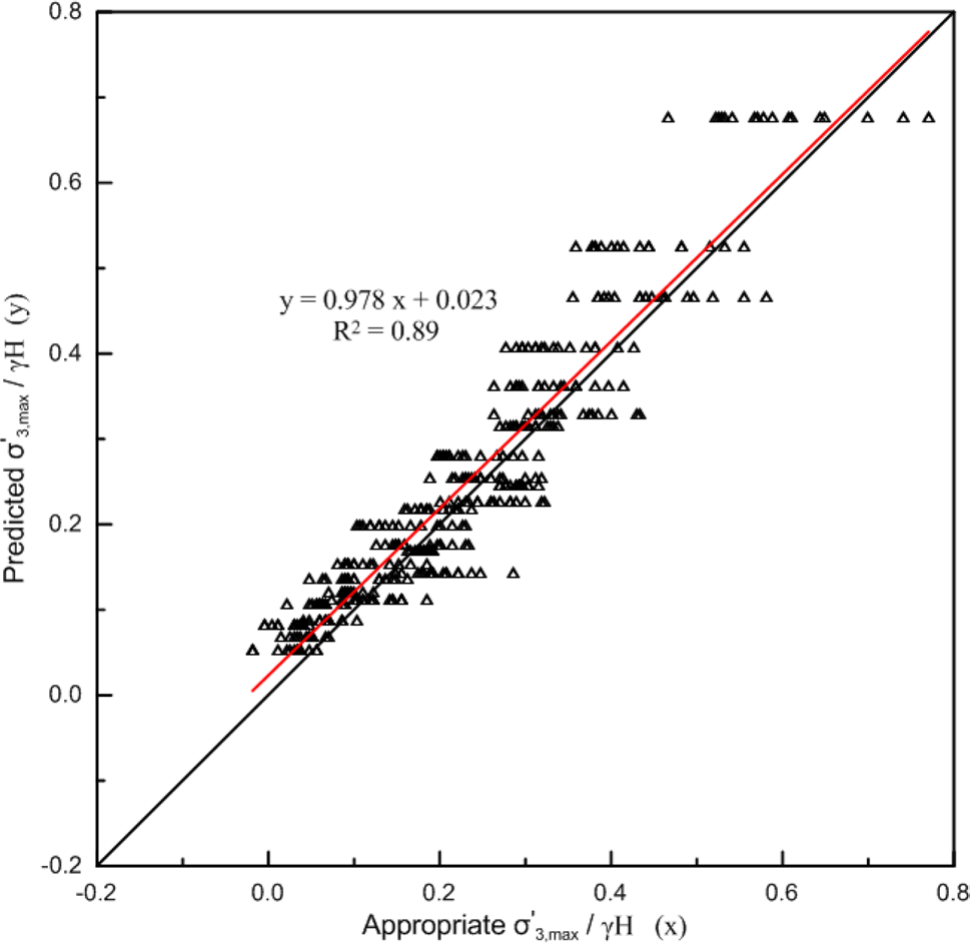
Comparison between appropriate $\frac{{\sigma_{3,\max }^{\prime } }}{\gamma H}$ values and predicted values from Eq. (17).

The relationship between $\frac{{\sigma_{3,\max }^{\prime } }}{\gamma H}$ estimated by Eq. (17) and the appropriate value is shown in Fig. 9.

Comparing Figs. 8 and 9, the prediction accuracy of Eq. (17) is slightly lower than that of Eq. (16), especially when $\frac{{\sigma_{3,\max }^{\prime } }}{\gamma H}$ is large, the dispersion of the predicted value using Eq. (17) increases.

**Figure 10:**
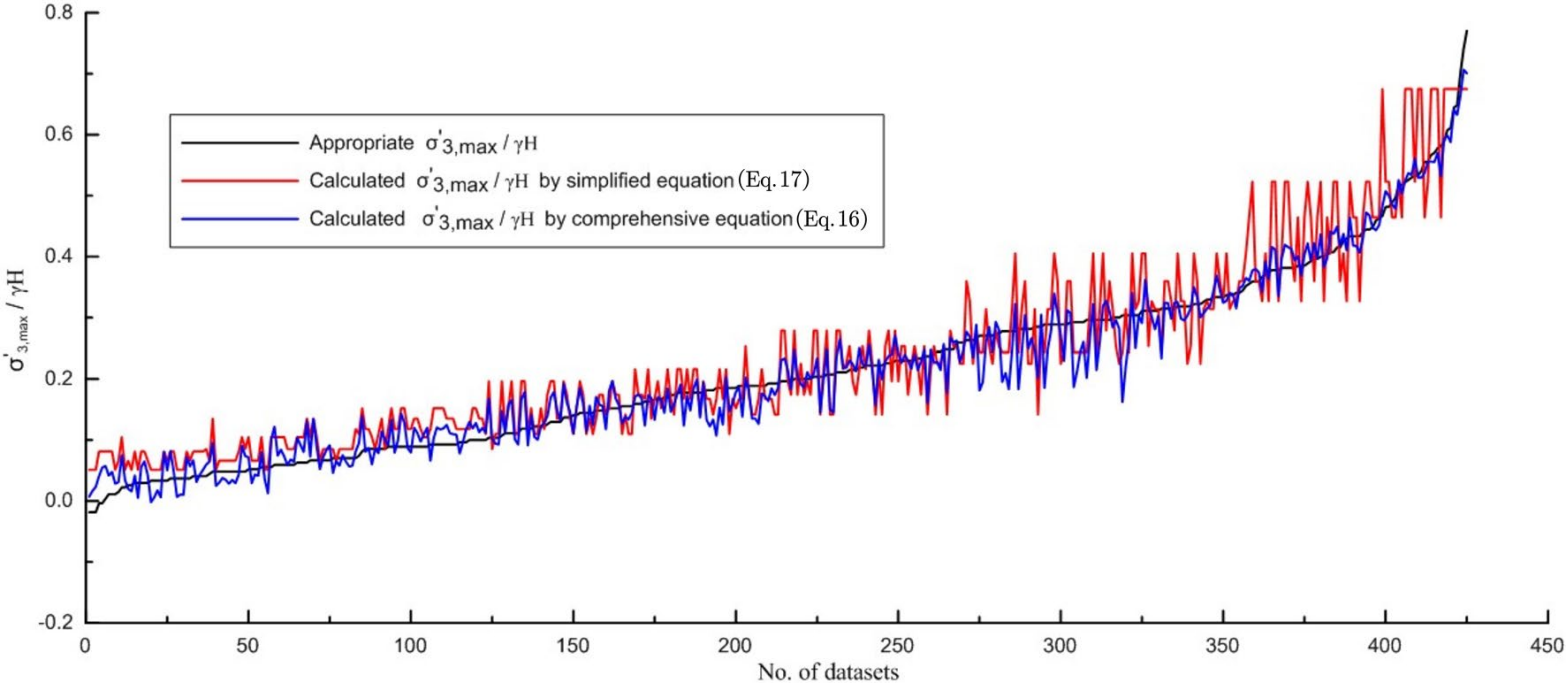
Predicted $\frac{{\sigma_{3,\max }^{\prime } }}{\gamma H}$ values from two quantitative correlations.

**Figure 11:**
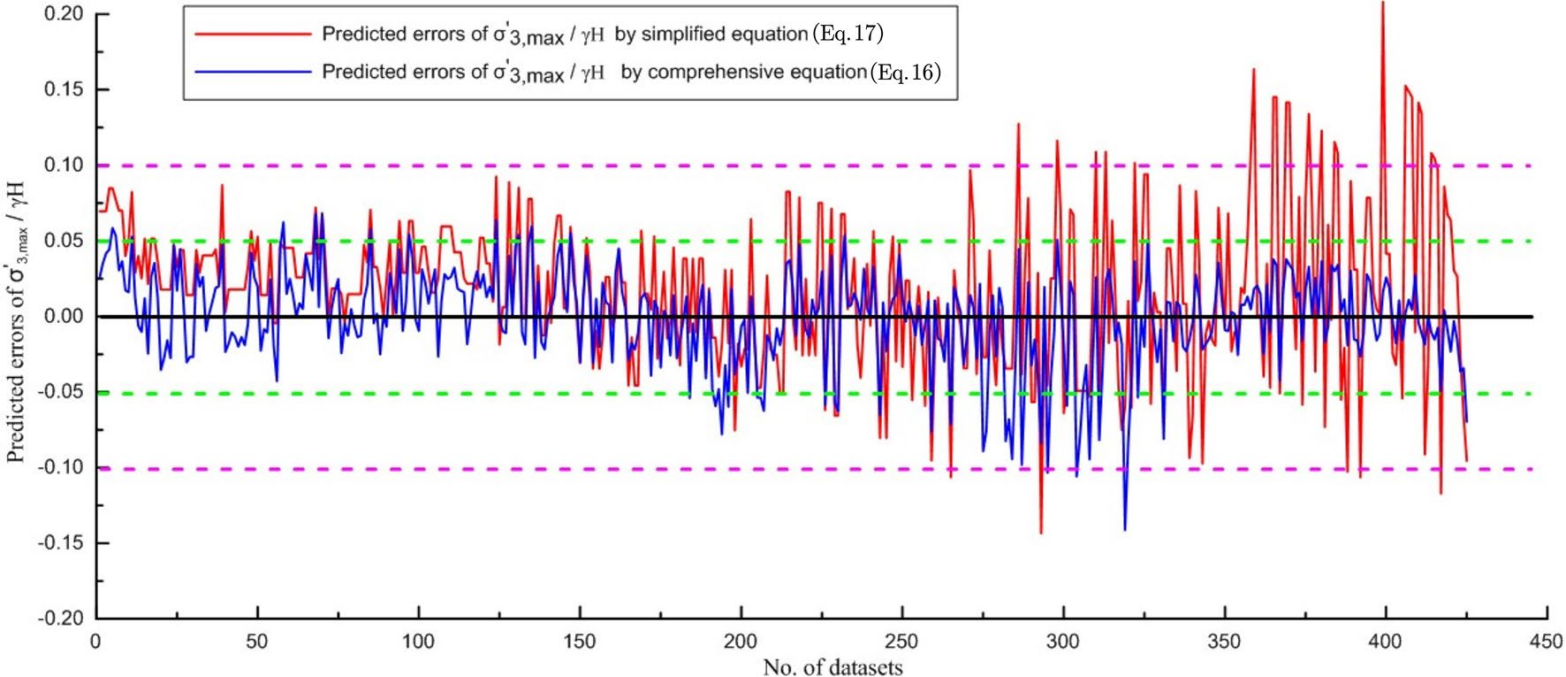
Predicted errors of $\frac{{\sigma_{3,\max }^{\prime } }}{\gamma H}$ values from two quantitative correlations.

In addition, for validating the simplified and comprehensive quantitative correlations, the $\frac{{\sigma_{3,\max }^{\prime } }}{\gamma H}$ predicted from Eqs. (16) and (17) are applied to comparing the appropriate $\frac{{\sigma_{3,\max }^{\prime } }}{\gamma H}$ value based on finite element method results. The predicted $\frac{{\sigma_{3,\max }^{\prime } }}{\gamma H}$ values are plotted and compared in Fig. 10. Predicted errors in $\frac{{\sigma_{3,\max }^{\prime } }}{\gamma H}$ from the simplified and comprehensive quantitative correlations are plotted in Fig. 11.

Most errors predicted by the comprehensive quantitative correlation (Eq. 16) are less than 0.05 (min − 0.141, max 0.068, average 0.020) and are smaller than those predicted by the simplified quantitative correlation (Eq. 17, min − 0.143, max 0.208, average 0.040), indicating Eq. (16) has the better performance (Figs. 10, 11).

Although the prediction accuracy of Eq. (17) is generally lower than that of Eq. (16), when $\frac{{\sigma_{3,\max }^{\prime } }}{\gamma H}$ is small (i.e., $\frac{{\sigma_{3,\max }^{\prime } }}{\gamma H}$ < 0.25), the prediction errors are equivalent. Only when $\frac{{\sigma_{3,\max }^{\prime } }}{\gamma H}$ is large (i.e., $\frac{{\sigma_{3,\max }^{\prime } }}{\gamma H}$ > 0.25) does the prediction error become significant (generally within 0.10). Therefore, when σ_ci_ and $m_{i}$ are not available, Eq. (17) can be used to estimate $\sigma_{3,\max }^{\prime }$.

It should be noted that Eqs. (16) and (17) are obtained from an analysis of slopes with the range of parameters given in Table 2, where apart from $\frac{{\sigma_{ci} }}{\gamma H}$ having limited range ($\frac{{\sigma_{ci} }}{\gamma H}$ = 10–50), the slope angle, GSI, and $m_{i}$, almost cover the complete possible value ranges.

## Validation using published data

In "New equation for estimating appropriate range of confining stress", two new equations for estimating appropriate range of confining stress have been established. To further validate the developed new equations, we have applied them to the various real cases of rock slopes^22^. The gathered data and calculation results are shown in Tables 3 and 4, respectively.

**Table 3:** various real cases of rock slopes^22^.

Cases	H (m)	*β* (°)	γ (kN/m^3^)	*σ*_*ci*_ (MPa)	GSI	*m*_*i*_	D
1	184	55	27	153	47	9	0.9
2	140	34	26	50	28	8	0.7
3	220	45	27	65	44	17	0.8
4	135	65	27	172	58	9	0.9
5	70	50	27	29	41	7	0.8
6	110	45	26.5	50	25	10	0.7
7	270	45	27	109	39	18	0.9
8	170	55	30	104	48	7	0.7
9	60	60	27	65	44	13	1
10	35	67	27	109	28	12	1
11	63	35	27	109	28	12	1
12	70	49	27	3	49	24	1
13	58	50	27	5	55	22	1
14	60	48	27	5	54	22	1
15	60	52	27	5	56	22	1
16	40	71	27	50	33	14	1
17	110	50	27	50	25	14	1
18	41	50	27	3	46	24	1
19	41	55	27	3	49	24	1
20	46	55	27	3	50	24	1
21	57	49	27	3	48	24	1
22	57	37	27	3	48	24	1
23	57	40	27	3	48	24	1
24	57	42	27	3	48	24	1
25	27	45	25	0.75	100	10	0
26	50	60	23	10	30	8	1
27	50	45	27	13.5	30	5	0.7
28	25	45	27	5.4	20	20	0.7
29	5	30	27	2.7	10	5	0.5
30	25	75	25	0.625	80	15	0.3
31	250	60	23	46	50	35	1

**Table 4:** Calculation results.

Cases	σ_ci_/γH	Appropriate $\frac{{\sigma_{3,\max }^{\prime } }}{\gamma H}$	Equations (10) and (11) $\frac{{\sigma_{3,\max }^{\prime } }}{\gamma H}$	Equation (12) $\frac{{\sigma_{3,\max }^{\prime } }}{\gamma H}$	Equation (16) $\frac{{\sigma_{3,\max }^{\prime } }}{\gamma H}$	Equation (17) $\frac{{\sigma_{3,\max }^{\prime } }}{\gamma H}$
1	30.80	0.1469	0.1896	0.0040	0.1808	0.1557
2	13.74	0.2747	0.4593	0.0189	0.2145	0.2386
3	10.94	0.1481	0.1988	0.0160	0.1478	0.2113
4	47.19	0.1591	0.1790	0.0017	0.1698	0.1250
5	15.34	0.1693	0.2017	0.0096	0.1738	0.1685
6	17.15	0.1544	0.2039	0.0102	0.1329	0.1530
7	14.95	0.1811	0.1988	0.0117	0.1411	0.1941
8	20.39	0.1765	0.1927	0.0060	0.1809	0.1584
9	40.12	0.0988	0.1876	0.0025	0.1302	0.1220
10	115.34	0.0317	0.1836	0.0006	0.0857	0.0684
11	64.08	0.2646	0.3543	0.0039	0.2624	0.2299
12	1.59	0.1005	0.2270	0.0958	0.0635	0.1999
13	3.19	0.1596	0.2134	0.0460	0.0981	0.2137
14	3.09	0.1358	0.2145	0.0511	0.1032	0.2255
15	3.09	0.0988	0.2133	0.0443	0.0908	0.2024
16	46.30	0.0185	0.1914	0.0013	0.0446	0.0604
17	16.84	0.1010	0.2112	0.0087	0.0931	0.1284
18	2.71	0.1716	0.2205	0.0542	0.0678	0.1834
19	2.71	0.1265	0.2187	0.0452	0.0558	0.1611
20	2.42	0.0966	0.2199	0.0507	0.0549	0.1638
21	1.95	0.1300	0.2244	0.0780	0.0669	0.1966
22	1.95	0.2469	0.5984	0.1191	0.1179	0.3001
23	1.95	0.2209	0.5984	0.1070	0.1036	0.2695
24	1.95	0.1949	0.5984	0.0997	0.0948	0.2511
25	1.11	0.2963	0.1996	0.1575	0.3019	0.5474
26	8.70	0.0348	0.2218	0.0116	0.0652	0.0962
27	10.00	0.1556	0.2141	0.0175	0.1650	0.1665
28	8.00	0.1481	0.2129	0.0219	0.0650	0.1404
29	20.00	0.2963	0.5135	0.0152	0.2222	0.2052
30	1.00	0.0480	0.2133	0.0469	0.0206	0.1043
31	8.00	0.0730	0.1995	0.0126	0.0539	0.1351

The root mean squared error (RMSE) is used as an indicator pf the misfit between the appropriate value and the predicted value.

18 $$RMSE = \sqrt {\frac{1}{N}\sum\limits_{i = 1}^{N} {\left( {\frac{{\sigma_{3,\max }^{{\text{p}}} }}{\gamma H} - \frac{{\sigma_{3,\max }^{{\text{a}}} }}{\gamma H}} \right)^{2} } }$$

where $\sigma_{3,\max }^{{\text{a}}}$ and $\sigma_{3,\max }^{{\text{p}}}$ are the appropriate and predicted value of $\sigma_{3,\max }$, respectively, and N is the number of cases. When an RMSE value approaches zero, the predicted values from the equation are closer to the appropriate values.

**Figure 12:**
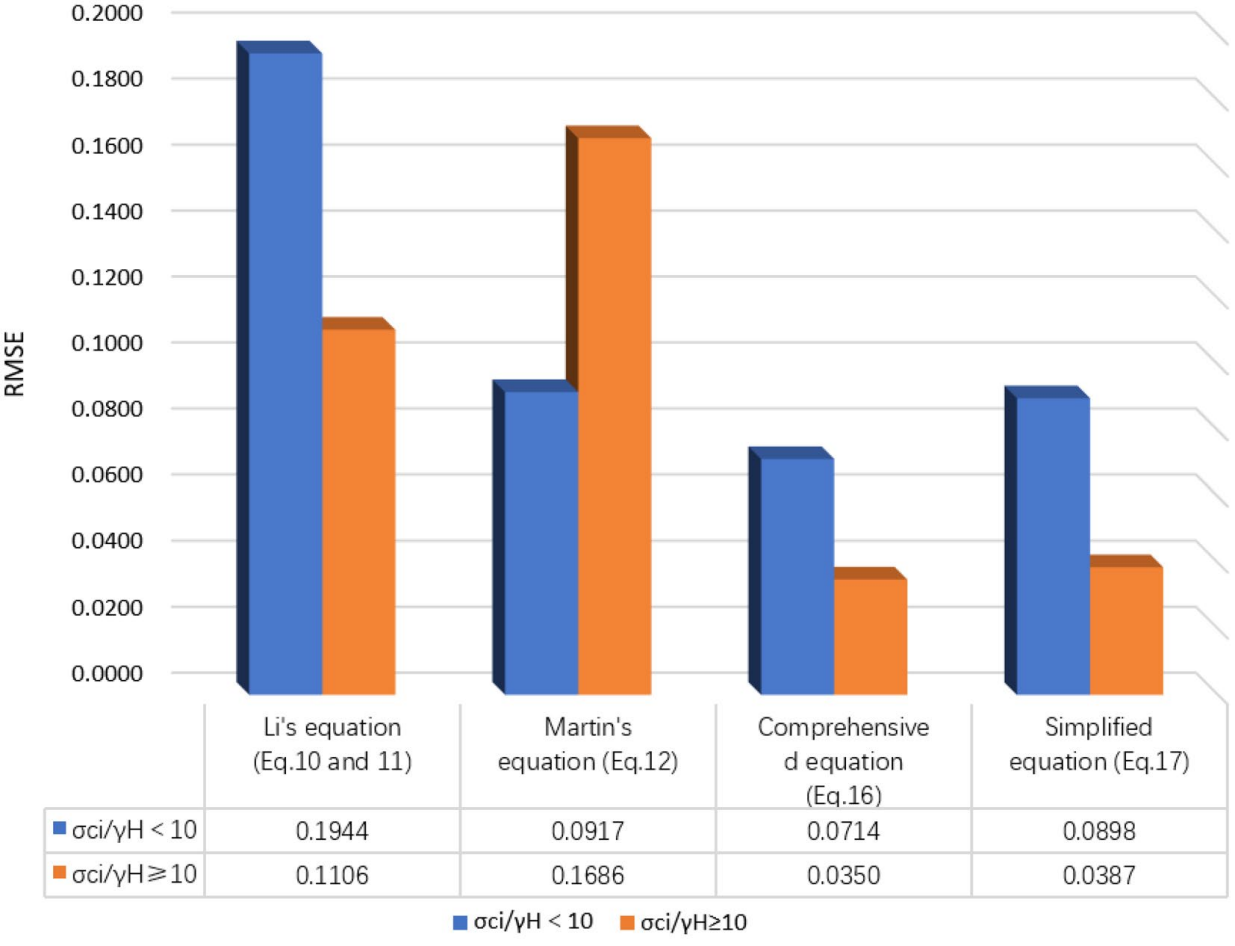
The RMSE obtained in the predicted $\frac{{\sigma_{3,\max }^{\prime } }}{\gamma H}$ value by different equations.

As can be seen in Fig. 12, the RMSE values form all modified predictive equations in this study are lower than the equations from previous studies.

**Figure 13:**
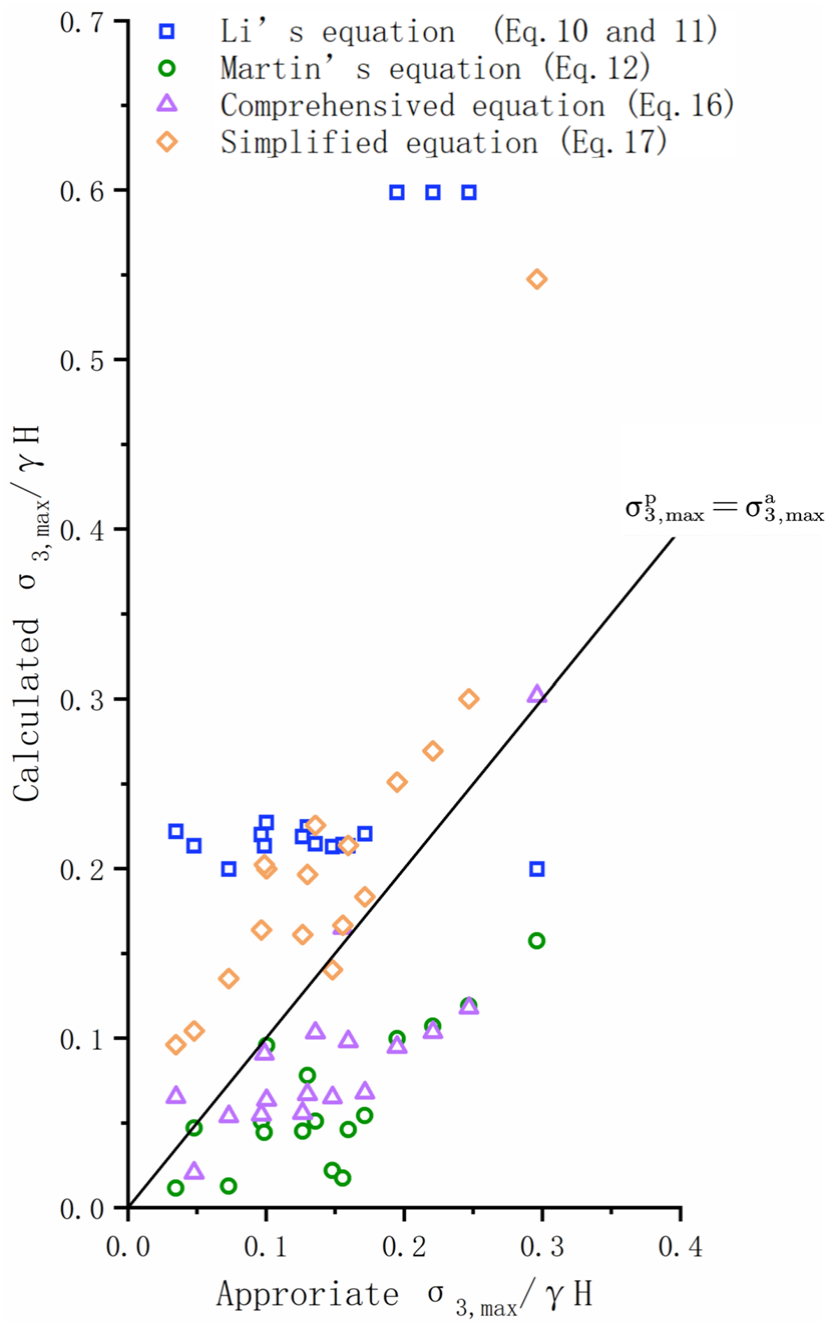
Comparison of the appropriate $\frac{{\sigma_{3,\max }^{\prime } }}{\gamma H}$ values and predicted values from different equations ($\frac{{\sigma_{{{\text{ci}}}} }}{\gamma H}$ < 10).

When $\frac{{\sigma_{ci} }}{\gamma H} < 10$, the predicted $\frac{{\sigma_{3,\max } }}{\gamma H}$ given in Eqs. (16) and (17) are both close to appropriate $\frac{{\sigma_{3,\max } }}{\gamma H}$. The predicted $\frac{{\sigma_{3,\max } }}{\gamma H}$ from Eq. (16) are smaller, and the predicted values of Eq. (17) are larger. The RMSE of the predicted value from Eq. (16) is 0.0714, and the Eq. (17) is 0.0898. The estimated $\frac{{\sigma_{3,\max } }}{\gamma H}$ given in Eqs. (10) and (11) are quite above the appropriate values with a mean absolute error of 0.1944. The prediction results of Eq. (12) are close to those of Eq. (16), but the accuracy is lower than that of Eq. (16) with RMSE of 0.0917 (Figs. 12, 13).

**Figure 14:**
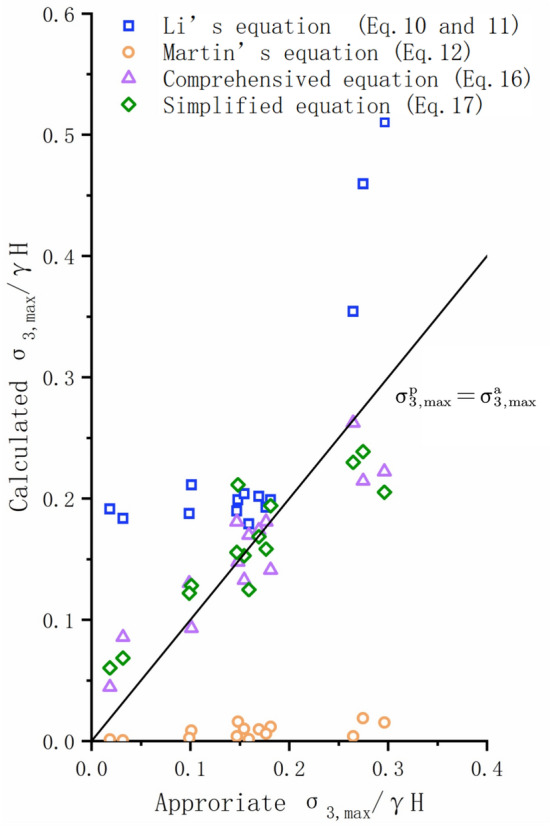
Comparison of the appropriate $\frac{{\sigma_{3,\max }^{\prime } }}{\gamma H}$ values and predicted values from different equations ($\frac{{\sigma_{{{\text{ci}}}} }}{\gamma H}$ ≥ 10).

When $\frac{{\sigma_{ci} }}{\gamma H} \ge 10$, the predicted $\frac{{\sigma_{3,\max } }}{\gamma H}$ given in Eqs. (16) and (17) are both very approach to the appropriate values. Meanwhile, the RMSE of the predicted value by Eqs. (16) and (17) are 0.035 and 0.0387, respectively. The predicted values of Eqs. (10) and (11) are higher than the appropriate values with RMSE being 0.1106. The prediction result of Eq. (12) is significantly smaller than the appropriate value with RMSE being 0.1686. (Figs. 12, 14).

## Conclusions

When the Mohr–Coulomb criterion is used to analyze slope stability, it is commonly necessary to use the Hoek–Brown criterion to obtain equivalent Mohr–Coulomb shear strength parameters, where the maximum value of the minimum principal stress on the potential failure surface of the slope is the most important parameter. Based on the finite element strength reduction method and elastic analysis, this paper systematically analyzes 425 different slopes. Slope angle and GSI have the most significant influence on $\sigma_{3,\max }^{\prime }$, followed by intact rock strength and *m*_*i*_. $\sigma_{3,\max }^{\prime }$ decreases with increasing slope angle and *m*_*i*_ as a power function and increases with increasing GSI and $\sigma_{{{\text{ci}}}} /\gamma {\text{H}}$ as an exponential and power function, respectively. On this basis, two new formulas for estimating $\sigma_{3,\max }^{\prime }$ are fit to the data, and the average errors of Eqs. (16) and (17) are 0.02 and 0.04, respectively, which shows the new formulas have good performance. To further validate the developed new equations, the equations were verified by 31 real slope cases. The result of verification shows estimated $\frac{{\sigma_{3,\max } }}{\gamma H}$ from Eqs. (16) and (17) are more consistent with the appropriate value than the predicted result from previous research, and the prediction accuracy of Eq. (16) compares well with others. The verification result indicating that new equations established in this contribution can be used in practical engineering.

## Data Availability

The datasets used and/or analysed during the current study available from the corresponding author on reasonable request.

## Abbreviations

$\sigma_{1}$: Major effective principal stress
$\sigma_{3}$: Minor effective principal stress
$\sigma_{ci}$: Unconfined compressive strength of intact rock
$\sigma_{cm}$: Unconfined compressive strength of rock mass
$\sigma_{t}$: Tensile strength of rock mass
$$\sigma_{v}$$: Gravitational stress
$\sigma_{3n}^{\prime }$: Normalized upper limit of confining stress
$\sigma_{3,\max }^{\prime }$: Upper limit of confining stress over the equivalent Mohr–Coulomb and Hoek–Brown criteria are considered
$\sigma_{3,\max }^{{\text{a}}}$: Appropriate value of $\sigma_{3,\max }$ obtained from elastic stress analysis
$\sigma_{3,\max }^{{\text{p}}}$: Predicted value of $\sigma_{3,\max }$
$m_{b}$: Hoek–Brown constant for rock mass
$m_{i}$: Hoek–Brown constant for intact rock
$s$: Hoek–Brown constant for rock mass
$a$: Hoek–Brown constant for rock mass
$$c\prime$$: Equivalent cohesion
$\phi \prime$: Equivalent friction angle
$GSI$: Geological strength index
$$D$$: Disturbance factor
$$\gamma$$: Unit weight
$$H$$: Slope height
$$\beta$$: Slope angle
$E_{rm}$: Deformation modulus of rock mass
